# Supplementary material for: Injuries among adolescents in Greenland: behavioural and socio-economic correlates among a nationally representative sample
Source: PeerJ. 2020 Feb 18;8:e8605. doi: 10.7717/peerj.8605 (PMC7034370; doi:10.7717/peerj.8605)
Supplement: Supplemental Information 2 [file peerj-08-8605-s002.docx]

**Appendix 1 (referred to as ‘Appendix 1’). Dependent and independent variable coding (2005/2006)**

| **Survey question** | **Response options** | **Recoded as** | **Variable** |
| --- | --- | --- | --- |
| Many young people get hurt or injured from activities such as playing sports or fighting with others at different  places such as the street or home. Injuries can include being poisoned or burned. Injuries do not include illnesses such as  Measles or the Flu. The following questions are about injuries you may have had during the past 12 months. How many times have you been injured in the past 12 months? | (1) I was not injured in the past 12 months  (2) 1 time  (3) 2 times  (4) 3 times  (5) 4 times or more | (0) I was not injured in the past 12 months  (1) 1-2 times  (2) 3 or more times | Injuries |
| What year were you born? What month were you born? | 1988-1998  Jan-Dec | Used as a continuous variable | Age |
| Are you a boy or a girl? | (1) Boy  (2) Girl | (0) Girl (1) Boy | Gender |
| Over the past 7 days, on how many days were you physically active for a total of at least 60 minutes per day? | (0) 0 days  (1) 1 day  (2) 2 days  (3) 3 days  (4) 4 days  (5) 5 days  (6) 6 days  (7) 7 days | (0) 3 days or less (1) 4 days or more | VPA |
| How often do you usually exercise in your free time so much that you get out of breath or sweat? | (1) Every day  (2) 4-6 times  (3) 2-3 times  (4) Once a week  (5) Once a month  (6) Less than once a month  (7) Never | (0) 3 days or less (1) 4 days or more | VPA outside school |
| How often do you smoke tobacco at present? | (1) Every day  (2) Once a week  (3) Less than once a week  (4) I do not smoke | (0) I do not smoke (1) Every day/Once a week/ Less than once a week | Smoking |
| Have you ever had so much alcohol that you were really drunk? | (1) No, never  (2) Yes, once  (3) Yes, 2-3 times  (4) Yes, 4-10 times  (5) Yes, more than 10 times | (0) No, 1-3 times (1) Yes, 4 times or more | Alcohol misuse |
| How often have you been bullied at school in the past couple of months? | (1) Haven’t  (2) Once or twice  (3) 2 or 3 times per month  (4) Once a week  (5) Several times a week | (0) Haven’t/Once or twice (1) 2 or 3 times per month/Once a week/Several times a week | Bullying victimisation |
| How often have you taken part in bullying another student(s) at school in the past couple of months? | (1) Haven’t  (2) Once or twice  (3) 2 or 3 times per month  (4) Once a week  (5) Several times a week | (0) Haven’t/Once or twice (1) 2 or 3 times per month/Once a week/Several times a week | Bullying perpetrator |
| During the past 12 months, how many times were you in a physical fight? | (1) I have not been in a physical fight  (2) 1 time (3) 2 times  (4) 3 times  (5) 4 times or more | (0) I have not been in a physical fight/1 time (1) Twice/3 times/4 times or more | Physical fighting |
| Occupational socio-economic status father | (1) SES 1  (2) SES 2  (3) SES 3  (4) SES 4 (5) SES 5  (6) In a job but unclassifiable  (7) Economically inactive, eg ill, retired, a student | (0) SES 5/In a job but unclassifiable/Economically inactive, eg ill, retired, a student  (1) SES 1-4 | Job father |
| Occupational socio-economic status mother | (1) SES 1  (2) SES 2  (3) SES 3  (4) SES 4 (5) SES 5  (6) In a job but unclassifiable  (7) Economically inactive, eg ill, retired, a student | (0) SES 5/In a job but unclassifiable/Economically inactive, eg ill, retired, a student  (1) SES 1-4 | Job mother |
| How easy is it for you to talk to the following persons about things that really bother you? (Friends of same gender) | (1) Very easy (2) Easy (3) Hard (4) Very hard (5) Does not have/see this person | (1) Very easy (2) Easy (3) Hard (4) Very hard (5) Does not have/see this person | Talk to friends  same gender |
| How easy is it for you to talk to the following persons about things that really bother you? (Friends of opposite gender) | (1) Very easy (2) Easy (3) Hard (4) Very hard (5) Does not have/see this person | (1) Very easy (2) Easy (3) Hard (4) Very hard (5) Does not have/see this person | Talk to friends  opposite gender |
| How well off do you think your family is? | (1) Very well off  (2) Quite well off (3) Average (4) Not very well off  (5) Not at all well off | (0) Not very well off/Not at all well off (1) Average (2) Very well off/Quite well off | Self-assessed wealth |
| Please answer this first question for the home where you live all or most of the time and tick the people who live there (Mother) | (1) Yes  (2) No | (1) Yes  (2) No | Live with mother |
| Please answer this first question for the home where you live all or most of the time and tick the people who live there (Father) | (1) Yes  (2) No | (1) Yes  (2) No | Live with father |
